# Identification of Two Genetic Haplotypes Associated with the Roan Coat Color in the American Quarter Horse and Other Equine Breeds

**DOI:** 10.3390/ani15121705

**Published:** 2025-06-09

**Authors:** Robin E. Everts, Rachael Caron, Gabriel Foster, Kaitlyn McLoone, Katie Martin, Samantha A. Brooks, Christa Lafayette

**Affiliations:** 1 Etalon Inc., Menlo Park, CA 94025, USA; rcaron@etalondx.com (R.C.); gfoster@etalondx.com (G.F.); kmcloone@etalondx.com (K.M.); khoefs@etalondx.com (K.M.); 2 Department of Animal Sciences, UF Genetics Institute, University of Florida, Gainesville, FL 32611, USA; samantha.brooks@ufl.edu

**Keywords:** horses, roan, KIT

## Abstract

The roan coat color is described as the dispersion of white hairs within an otherwise solid background color coat. This phenotype is primarily expressed on the body of the horse, with the head and legs exhibiting few to no white hairs. Previous studies mapped the locus for roan to the KIT region and observed linked variants in a small number of breeds. However, utilizing those linked markers to determine the roan genotype in other breeds has seen limited success. In this communication we identify a second roan allele (RN2) which, in conjunction with a previously observed roan allele (RN1) discovered in previous studies, accounts for approximately 74%, or 188 horses, out of a sample size totaling 257 roan horses. These two alleles were present in the non-roan population (N = 3212) at less than 1%, only in horses with light coat color and dilution alleles, likely obscuring the roan phenotype. Future work is required to identify additional alleles responsible for additional roan-type horse coat color phenotypic variation.

## Simple Summary

The roan coat color is described as the dispersion of white hairs within an otherwise solid background color coat. This phenotype is primarily expressed on the body of the horse, with the head and legs exhibiting few to no white hairs. Previous studies mapped the locus for roan to the *KIT* region and observed linked variants in a small number of breeds. However, utilizing those linked markers to determine the roan genotype in other breeds has seen limited success. In this communication, we identify a second roan allele (RN2) which, in conjunction with a previously observed roan allele (RN1), accounts for approximately 74%, or 188 horses, out of a sample size totaling 257 roan horses. Within the non-roan population (N = 3212) these alleles were present at less than 1% total, mostly in horses with light coat colors due to dilution variants. Future work is required to identify additional alleles responsible for additional roan-type horse coat color phenotypes.

## 1. Introduction

Roan is inherited as a dominant trait and is epistatic to other coat colors, meaning the base coat color is expressed but will contain white hairs dispersed throughout the regular coat color on the animal’s body [1]. The roan coat color has also been observed in other livestock species and is likely associated with the KIT or KITLG gene [2]. In horses, variants within the KIT locus were previously linked to the roan phenotype in work published in 1982 [3,4] and more recently in the Noriker horse [5] and Icelandic horse [6]; yet, no definite causative variant(s) for the roan phenotype has been described. Several SNPs were identified for use as tag SNPs for the roan coat color in these two breeds. In the Noriker horse, the SNP chr3:79,543,439 A > G was statistically associated with the roan phenotype, and in the Icelandic horse, an insertion (chr3:79,548,355insC) possessed the strongest correlation to roan (all coordinates per EquCab3.0; [7]). Grilz-Seger et al. (2020) [5] identified the roan phenotype in a few additional breeds but that study also had a small sample size. In addition to roan, other polymorphisms in the KIT gene cause different white spotting phenotypes in horses, such as Tobiano, Sabino, and Dominant White variants [8,9]. As many breeders use coat color as part of their breeding decision, being able to take into account the roan genotypes helps these breeders to more clearly select horses that will provide the expected outcome.

## 2. Materials and Methods

Genotype and coat color data were collected from our database for an initial case-control sample set of roan (N = 25) and non-roan (N = 30) horses that were processed by our standard workflow [9]. In short, genomic DNA was extracted from hair samples using the Puregene Extraction Kit following the manufacturer’s protocol (QIAGEN, Inc., Germantown, MD, USA), or retrieved from previously stored and extracted gDNA, where applicable, and subsequently sequenced on the commercially available platform of Etalon, Inc., with 2 × 150 bp reads on a NextSeq1000 instrument (Illumina, San Diego, CA, USA). The resulting sequences had base call quality scores ≥ Q30 and read depth >40× for all regions under investigation after alignment to EquCab3.0. Next, we used IGV version 2.16.2 [10] to screen the resulting bam files for the presence of variants in the KIT region on chromosome 3 that potentially distinguished roan and non-roan samples in the case-control dataset under the assumption *roan* is a dominant trait.

The candidate variants were validated using the Fisher’s Exact test in a larger cohort of 3469 horses consisting of 257 roan horses and 3212 non-roan horses. Candidate variants passing the statistical threshold were then analyzed for putative co-segregation and assigned into “roan haplotypes”. All genotype data for these horses was previously obtained using the NGS process as detailed above.

For the roan horses, coat color for most horses was confirmed by either owner-provided pictures, reaching out to the owner for information, or checking online pedigree tools like allpedigrees.com. For non-roan horses no outreach was performed unless genotype data needed clarification.

## 3. Results and Discussion

Using the limited data set of the 25 roan and 30 non-roan horses, we identified 30 variants that segregated differently between roan and non-roan horses. For these variants genotype data was then collected from a larger cohort (*n* = 3469) of horses for which phenotypic data specific to coat colors was available. From this larger set, 257 were roan horses and 3212 were non-roan horses based on the owner-reported coat colors. Tests of association for the 30 candidate variants using Fisher’s exact test revealed that 19 loci were not linked to the roan phenotype in this larger sample set and thus were discarded (all *p* > 1^−10^ with odds < 6). Additionally, the allele frequencies of the excluded variants were in excess of 3% in both roan and non-roan samples. The remaining 11 variants showed high prevalence in roan samples, over 35%, when compared to non-roan samples where they were present only in <1% of the horses, except for assays chr3:79588129 and chr3:79623598, which were present at 5% and 38%, respectively. These 11 assays were strongly associated with the phenotype (all *p* < 10^−15^ with odds of >20, Fisher’s exact test) and segregated into two likely roan haplotypes (Table 1). Both roan haplotypes had *p* < 10^−15^ with odds of >60 (Fisher’s exact test). As we distinguished two clearly independently segregating alleles we opted to differentiate these by calling them haplotypes Roan 1 (RN1) and Roan 2 (RN2), like how Dominant White alleles in the KIT region are being labeled with sequential allele numbering [8]. 

The roan haplotype termed Roan1 or RN1 was present in 99 (38%) of the tested roan-samples. This haplotype is comparable to the one found by Grilz-Seger et al. (2020) [5] and was found present in multiple breeds such as American Quarter Horse, American Paint Horse, Mustang, Gypsy Vanner, Welsh Pony and Cob, Mangalarga Marchador, Percheron, Belgian Draft, European Brabant, Missouri Fox Trotter, and Curly Horse. Therefore, this haplotype can be considered a likely ancestral allele (Table 2). Within the Quarter Horse, we looked at the extended pedigrees of several horses (https://www.Allbreedpedigree.com (accessed on 15 March 2025)), and assuming a dominant trait, we found this to be the roan haplotype traced to the Burnett roan mare (born 1901, AQHA registry U0069050). One major Quarter Horse sire likely contributing this roan haplotype to the modern breed is Red Man (born 1935). We counted 88 RN1 heterozygous, seven homozygous RN1/RN1, and two RN1/RN2 horses within this group.

For the Roan2 (RN2) allele, present in 93 (36%) of the tested roan-samples, none of the variants were described previously. This allele is present in fewer breeds, all based on North American ancestry including the American Quarter Horse, Mustang, Curly Horse, and American Paint Horse (Table 2). Within the Quarter Horse, using the same pedigree analysis as described for Roan1, we found this to be the roan haplotype that can be traced to the Burns roan mare (born 1901, AQHA registry U0069059) and her offspring Orals Kitten (born 1935, AQHA registry 0037735). This seems a newer haplotype as it is present only in breeds that originated in the US. It is currently likely propagated by the influential sires Metallic Cat (born 2005) and Peptoboonsmal (born 1992). We counted 87 RN2/n, 2 homozygous RN2/RN2, and 2 RN1/RN2 horses within this group.

Together these two alleles cover about 74% of the roan samples tested. Yet 65 (25%) horses that were labeled roan did not possess any novel variants in the KIT region interrogated, which would suggest the existence of other alleles or phenocopies produced by another locus. See Table 3 for these unknown roan horses. Thus, it is likely that there remain additional roan variants yet to be discovered. The sensitivity of the roan test using the two haplotypes identified here is about 68%, and the specificity is approximately 99%, meaning these markers identify roan phenotypes specifically. The sensitivity is reduced as the current roan markers are absent in approximately 25% of the tested roan samples, but present in a small fraction of horses described as not possessing a roan phenotype. For a subset of purebred horse breeds with unknown roan alleles see Table 3. We also confirm that homozygous roan diplotypes show no present indication of lethality as our sample set possessed seven homozygous RN1/RN1 samples, two homozygous RN2/RN2 samples, and two compound heterozygous RN1/RN2 roan samples.

Among the non-roan samples, we counted 27 horses (<1%) that tested positive for a roan allele. Of these, 22 were positive for the RN1 haplotype and the remaining five were positive for the RN2 haplotype. Many of these horses were described by the owner as another non-roan coat color, such as gray, buckskin, or grullo, or had at least one of the dilution variants causing a very light coat color. Thus, for many of these horses, another coat color locus may obscure the presence of the roan phenotype.

## 4. Conclusions

Overall, the RN1 allele was present in 3%, the RN2 allele in 3%, and the unknown allele(s) in 2% of all the horses. As we report on two roan alleles but are still missing about 25% of known roan samples, we expect at least one more roan allele to be present in the modern horse population. In additional sample sets, we observed that approximately 6% of horses carried at least one of the two roan alleles, and we were able to correctly identify around 60% of the owner-identified and visually verified roan samples. We would like to emphasize that these haplotypes are based on association only and are not likely to include the causal mutation. Yet, this work is the first use of these two haplotypes to detect roan alleles in a broad set of breeds and to assess this association in a large sample size. Further research into the genetic basis of the collection of roan phenotypes needs to be performed and is ongoing. The fact we identified two completely independent haplotypes associated with the roan coat color implies there may be multiple origins for roan. As current studies have used roan as one homogeneous group this may have been a reason for the difficulty in ascertaining the cause(s) of the roan coat color. As the study by Grilz-Seger et al. (2020) [5] already used the 670K SNP array, the most likely road to success would be analysis of whole genome sequencing of homozygous roan horses (by haplotype) vs non-roan horses.

## Figures and Tables

**Table 1:** Identified roan alleles and their respective variants included.

Roan Alleles *	Allele 1 (Ref)	Allele 2 (RN)	AF ^†^ Allele 2 (Rn/Non-Rn)	Accession Number
RN1/Roan1				
chr3:79533215–79533222	TCTCGTCT	DEL	0.370/<0.01	rs3433250193
chr3:79533282–79533285	TTCT	DEL	0.370/<0.01	rs3433767139
chr3:79543439	A	G	0.370/<0.01	rs1136290549
chr3:79588129 **	A	T	0.394/0.05	rs3435286779
chr3:79540501	G	A	0.370/<0.01	rs1140417535
chr3:79472198	T	A	0.352/<0.01	rs1152351721
RN2/Roan2				
chr3:79537494	T	C	0.373/<0.01	rs3435658556
chr3:79548451	G	A	0.391/<0.01	rs3435587371
chr3:79660650	C	A	0.384/<0.01	rs3432423278
chr3:79689294	A	G	0.384/<0.01	rs3433571795
chr3:79693449	C	T	0.384/<0.01	rs3431088654

* all positions on EquCab3.0. ** proxy for the ECA3:79,588,128–79,588,130delinsTTATCTCTATAGTAGTT. ^†^ Allele frequencies of the respective roan alleles in the roan sample set and the non-roan sample set.

**Table 2:** Prevalence of the two roan alleles in different horse breeds. Does not contain mixed breed animals.

RN1		RN2	
Breed	# Horses	Breed	# Horses
American Quarter Horse	43	American Quarter Horse	73
Gypsy Cob/Vanner	18	American Paint Horse	8
Mustang	5	Mustang	2
American Paint Horse	5	Curly Horse	2
Draft Cross	3	Mixed Breed	8
Mangalarga Marchador	3		
Percheron	3		
Tennessee Walking Horse/Tennessee Walker	3		
Nokota	2		
Welsh Pony	2		
Criollo/Crioulo	1		
Irish Cob	1		
Mangalarga	1		
Missouri Fox Trotter	1		
Spanish-Barb	1		
Curly Horse	1		
Mixed Breed	6		

**Table 3:** Prevalence of the unknown roan horses in different breeds. Does not contain mixed breed animals.

Breed	# Horses
American Quarter Horse	24
American Paint Horse	17
Appaloosa	5
Tennessee Walking Horse/Tennessee Walker	2
Gypsy Cob/Vanner	2
Mustang	1
Unknown	1
Curly Horse	1
Irish Cob	1
Pony of the Americas	1
Thoroughbred	1
Walkaloosa	1

## Data Availability

Horse sequencing data are not available due to owner confidentiality.
